# Specialized core bacteria associate with plants adapted to adverse environment with high calcium contents

**DOI:** 10.1371/journal.pone.0194080

**Published:** 2018-03-08

**Authors:** Fei Li, Ximin Zhang, Jiyi Gong, Lunxian Liu, Yin Yi

**Affiliations:** Key Laboratory of Plant Physiology and Developmental Regulation, School of Life Sciences, Guizhou Normal University, Guiyang, Guizhou, China; Estacion Experimental del Zaidin, SPAIN

## Abstract

Karst topography is formed from the dissolution of soluble rocks, such as limestone and dolomite. In soils of such a landform, excessive contents of exchangeable calcium seriously limit the growth of vegetations. Researches have proved that rhizosphere microorganisms and endophytes help host plants to adapt to various adverse environments. The adaptive capacity of plants that grow in adverse environment with salt, drought, thermal and heavy metal stresses partially or completely comes from symbiotic microorganisms. By using the high-throughput amplicon sequencing, the bacterial community structures in soil with high calcium contents and roots and leaves of *Cochlearia henryi* that is commonly seen in karst area were analyzed. The bacteria community structures in these three compartments showed obvious differences. This indicates that *C*. *henryi*, which is adaptive to high calcium stress, selectively co-exists with specific bacteria. Although the bacteria community structures in these three compartments differed significantly, there were 73 operational taxonomic units (OTUs) shared by karst soils as well as roots and leaves of *C*. *henryi*. The phylogenetic diversity of these 73 OTUs differed significantly from that of overall OTUs detected. There were also obvious differences in KEGG (Kyoto Encyclopedia of Genes and Genomes) pathways and abundance values between the 73 OTUs and overall bacterial communities. A large number of OTUs shared by the karst soils, roots and leaves of *C*. *henryi* had close genetic relationship with known stress-resistant bacterial strains. Our results showed that the functional bacteria can be predicted by exploring core bacteria, bacteria shared by soils, adaptable plant roots and leaves. This information will potentially accelerate studies on natural microbial communities which can promote the adaptive capacity of host plants to high calcium stress, and will be valuable for finding microbial strains for field application in karst topography.

## Introduction

Rhizosphere microorganisms and endophytes can help host plants to adapt to various adverse environments [1]. Soil property is the critical factor determining the community structures of plant rhizospheric and endophytic microorganisms [2,3]. Exploring and screening regional adaptive microorganisms is an important way to develop microbial fertilizer, so as to realize sustainable development of regional agriculture.

The karst region centered on Guizhou province in the southwest China is the largest karst topographic region in the world, covering more than 550,000 km^2^. Soils in karst region show two obvious characteristics. The first one is the high content of exchangeable calcium ions, reaching 1%~3%, a percentage several times higher than that of the soils in non-karst regions. The other one is the extremely poor soil and water conservation capacity subjecting the soil surface to severe drought [4].

The intake of calcium by plants is directly related to the contents of exchangeable calcium in soils. High calcium contents can result in excessive calcium ions intake, causing serious damages, including hardened cell walls, cell growth inhibition and disturbance of energy metabolism. Moreover, excessive calcium intake causes damages to plant cell membrane, which decreases the photosynthetic and transpiration rates, resulting in leaf senescence [5, 6]. Therefore, plants growing in karst environment have to possess unique physiological adaptation mechanisms.

Recent experimental evidences have proved that the adaptive capacity of plants to adverse environment with salt, draught, thermal, or heavy metal stresses is partially or completely attributed to symbiotic microorganisms [7–12]. Greenhouse and field experiments have demonstrated that some stress tolerant plants are unable to adapt to their original habitat if key endophytes are removed [7]. At present, various endophytic microorganisms, such as bacteria, fungi, mycorrhizal fungi, viruses and microalgae have been found in all analyzed plants [9, 10, 13]. These microorganisms exist between cells, in xylem vessels, and even in cells of all plant organs, including roots, stems, leaves, flowers, fruits and seeds [14, 15]. Endophytic strains isolated from geothermal habitat in Yellowstone National Park (the Unites States), salinization habitat in seabeach and farmland habitat, can infect dicotyledonous tomatoe and monocotyledonous rice, and these inoculated plants are endowed with resistance against heat, salt and disease stress, respectively [8]. The drought treatment of *Brassica rapa* for three successive generations shows that the drought resistance ability of *B*. *rapa* offspring does not change significantly. However, the soils treated with drought stress can help plants growing in it to adapt to drought environment, indicating that microorganisms in soils evolve more rapidly to adapt to adverse environment than plants [16]. Some scientists propose that the phenotype of plants in the nature is a product of the synergistic interaction between the plant genome and microorganisms that widely exist in the rhizosphere or endosphere of plants [15]. *Cochlearia henryi*, a sibling species of *Arabidopsis thaliana*, is commonly seen in Huajiang karst region of Guizhou province (China). Microorganisms in the karst soils faces calcium stress and endophytes in the roots and leaves of *C*. *henryi* must adapt to their host. However, data on the physiological and ecological effects of *C*. *henryi* rhizosphere microorganisms and endophytes in karst regions, a unique ecological system with high calcium content, are unknown, if not, limited and needs extensive scrutiny.

In the present study, in order to find out bacteria species that are widely present in karst ecosystem that are associated with *C*. *henryi*, three compartments, namely karst soils and roots and leaves of *C*. *henryi* were collected for high-throughput amplicon sequencing. “Core” operational taxonomic units (OTUs) shared by karst soils, as well as roots and leaves of *C*. *henryi* were identified. We expected that these OTUs should be adaptive to calcium stress and involved in the adaptability of *C*. *henryi* to karst ecosystem. To test this hypothesis, we compared the phylogenetic diversity and functional profiling of the “core” OTUs and that of the overall OTUs and observed significant differences.

## Materials and methods

### Field sampling

*C*. *henryi* is widely distributed in southwestern karst regions of China. In July, 2016, the *C*. *henryi* seedlings (with average height of 20 cm) growing in karst soils in the remote mountain of Huajiang county, Guizhou province, China (N25°43'21”, E 105°37'24”) were collected. No specific permissions were required for the field sampling and this field study did not involve endangered or protected species. In order to avoid potential sampling deviations caused by soil heterogeneity, two sample plots were selected with a distance of about 3~5 m. Three *C*. *henryi* seedlings (average height of about 20 cm) per plot were randomly collected, as biological replicates from each sample plot. The samples were divided into three parts, including soils, *C*. *henryi* roots and leaves. All samples were placed into 10 °C cold storage containers and then quickly transferred to the laboratory for further treatment.

### Soil analysis

The soil samples from the control nutrient soils and Karst bulk soils from sample plot 1 and sample plot 2 were used for analysis. The nutrient soil samples, used as a control for comparison, were for greenhouse use and were bought from Hunan Xiang Hui Agricultural Technology Development Co.,Ltd. To collect soils, an auger drilling machine was used for sampling 20 cm underground soils. The soils were grinded into particles with a mortar and then dried for 3 h at 120 °C for determining the soil composition. After drying, soil samples were filtered with a 2-mm sieve and used for the analysis of chemical and physical properties. The pH of soil samples was determined with a pH-meter using soil solutions (soil:water ratio of 1:5) obtained by shaking suspensions at 20 °C for 2 h on a shaker. Exchangeable calcium (Ca), sulfate (S), potassium (K) and magnesium (Mg) were extracted with 1N CH3COONH4 at alkaline pH = 9 and detected using the Atomic Absorption Spectroscopy approach. Total soil N was determined based on a simplified version of the Kjeldahl method [17]. Nitrogen fractions (NH4 and NO3) were extracted with 1 M KCl and analyzed by flow injection analysis. Total carbon in soil samples were determined by using the wet combustion method [18]. Phosphorus (P) was extracted in 0.5 mol/L sodium bicarbonate at pH 8.5 and determined by performing the flow injection analysis. A solution of DTPA containing, 0.01 M CaCl2, 0.1 M TEA and 0.005 M DTPA, pH = 7.3) was used for extracting the available forms of Aluminium (Al), copper (Cu), iron (Fe), sodium (Na), manganese (Mn) and zinc (Zn) prior to the determination of their concentrations using the Atomic Absorption Spectroscopy.

Experiments were performed in triplicate.

### Method for eliminating epiphytic microorganisms on plant tissues

The plant tissues (roots and leaves) were soaked in ethanol (75% vol/vol) for 40 seconds and then in 1% (vol/vol) sodium hypochlorite for 4 minutes and finally rinsed in sterile distilled water for 3 times. To verify the sterilization effectiveness, the sterile water used for washing was plated and cultivated on LB medium. The study continued only when no bacterial colony appeared on the LB medium, which indicates that surface sterilization was successful. The sterilized plant tissues were then immersed in the commercial DNA AWAY solution for 5 minutes and then washed 3 times with sterile water.

### DNA extraction

DNA was extracted from the roots and leaves of *C*. *henryi* by using the DNeasy Plant Mini Kit (QIAGEN). DNA was extracted from soil samples by utilizing the Power Soil DNA Isolation Kit (MOBIO). The samples were grinded in liquid nitrogen using sterile mortars and pestles frozen in advance. About 100 mg plant samples were utilized for DNA extraction.

### PCR amplification and pyrosequencing

Fourteen amplicon sublibraries were established for 454-pyrosequencing, namely two karst bulk soils, three *C*. *henryi* roots and leaves from each of the two sampling plots. OTUs that repeatedly appeared in these fourteen amplicon libraries were regarded as “core OTUs”.

The amplification primers for bacterial 16S rRNA variable region were 515F-806R primer (515F: GTG CCA GCM GCC GCGGTAA and 806R: GGA CTA CHV GGG TWT CTA AT, V4 variable region). This primer pair has been proven useful in reducing the contamination caused by amplification of plant sequences. Then, the 454 GS FLX + platform (Roche) in Beijing Institute of Genomics (Shenzhen, China) was employed for gel purification and sequencing of the amplicons. The amplicon sections were recovered, and the cohesive ends were repaired into flat end through T4 DNA Polymerase, Klenow DNA Polymerase and T4 Polynucleotide Kinase. Then, the nucleotide “A” was added to 3' end, so that amplicons can be connected to adaptors with “T” nucleotide in 3’ end. The forward primers included Adaptor A of 454 fusion primer (CCATCTCATCCCTGCGTGTCTCCGAC), 4bp library key sequence (TCAG), 10-bp multiplex identifier (MID) (AGCGTCGTCT) and primer sequences. The reverse primers consisted of Adaptor B (CCTATCCCCTGTGTGCCTTGGCAGTC) and specific primer sequences.

### Sequencing quality control and data analysis

In the processes of sequencing and data analysis, multiple quality control methods were used. The sequencing quality was inspected using Mothur software (v1.31.2) [19]. The trim.seqs command was used and the specific operations were as described below. The sequences were trimmed when the average quality score over a 30bp window dropped below 20. The sequences with final length lower than 75% of the original length were equally trimmed. The sequences with undetermined nucleotide, more than 8 continuous same nucleotide or joint contamination were deleted. By utilizing Mothur software unique.seqs. command, the non-redundant sequences were obtained. The UCHIME software version 4.2 (http://drive5.com/uchime) was employed to identify and delete chimera. The software FLASH (Fast length adjustment of short reads, v1.2.11) was used for sequence matching. Based on overlapping sequences, pairs of reads obtained through paired-end sequencing were assembled into a Tag. The Tags showing 97% or greater similarity were clustered into an OTU using the software USEARCH (v7.0.1090). The OTU sequences were compared with database for species annotation using RDP classifier software v2.2 [20] and the confidence threshold was set to be 0.8. All OTUs that were annotated as plant plasmid or mitochondrion 16S rRNA sequences were removed. The databases were Greengene (V201305) and RDP (Release9 201203). The classified sequences per taxa and per sample generated with both databases were counted at each rank and normalized with the number of classified sequences at a given rank.

### Statistical analysis

To evaluate the degree to which our sequencing coverage captured sequence diversity in each sample, we conducted rarefaction analysis. The tags were randomly resampled at various depths to simulate the effects of lower sequencing coverage. For each simulated sequencing depth, we randomly sampled with replacement and counted the number of OTUs identified in the sampled subset. Sampling was performed 500 times for each simulated sequencing depth to calculate the average number of OTUs detected at each depth. The rarefaction analysis was done with mothur program (version 1.31.2) (S1 Fig).

The non-metric multidimensional scaling (NMDS) was analyzed using the vegan package in R (v3.3.2). The Venn diagram showing the shared or specific OTUs of each sample or compartment was generated using the Venn Diagram package in R (v3.3.2), The core OTUs shared by the high calcium soil, roots and leaves of *C*. *henryi* were screened in accordance with the results of Venn diagram. The phylogenetic relationships of OTUs obtained by comparison with the databases were used to draw the abundance histogram of bacteria community species. For OTUs commonly existing in high calcium soil, roots and leaves of *C*. *henryi*, the heat map analysis was conducted according to the relative abundance of each OTUs in each sample.

The online Phylogenetic Investigation of Communities by Reconstruction of Unobserved States (PICRUSt) tool (http://huttenhower.sph.harvard.edu/galaxy/root?tool_id=PICRUSt_normalize) was used for PICRUSt analysis following the software instructions step by step. Firstly, we created the OTU table compatible with PICRUSt, which was normalized by the predicted 16S rDNA number. Then the metagenome predictions were obtained.

## Results

### Karst bulk soils in Huajiang county are alkaline and infertile with high calcium content

To determine the characteristics of soils in Huajiang county, the composition data of soil samples from the control nutrient soils (for greenhouse use) and karst bulk soils from sample plot 1 and sample plot 2 were determined. As shown in S1 Table, karst bulk soils were alkaline (pH value of 7.68 and 8.02 for soil samples from sample plot 1 and sample plot 2, respectively) and the content of exchangeable calcium was nearly 10 times that of nutrient soils (2.28% for karst bulk soil from sample plot 1 and 1.98% for karst bulk soil from sample plot 2 versus 0.24%). However, the organic nutrient substances, such as carbon and nitrogen which are important components for plant growth were lower than those of nutrient soils, showing a considerable degree of infertility. These results indicated that karst bulk soils in Huajiang county are alkaline and infertile with high calcium content, which points to a functional role of determinant factors, such as symbiotic bacterial community, in plant growth in these soils.

### Characteristics of bacterial community of karst bulk soil and *C*. *henryi* roots and leaves

Bacterial 16S amplicon sequencing was carried out on soil samples as well as roots and leaves of *C*. *henryi* from the two sample plots. Rarefaction analysis indicated that our sequencing coverage efficiently captured sequence diversity in each sample (S1 Fig). The abundance of OTUs in karst high calcium soils was largely greater than those of *C*. *henryi* roots and leaves. Similarly, the number of OTUs in roots and leaves of *C*. *henryi* showed obvious differences. There were fewer OTUs in *C*. *henryi* leaves comparatively to root samples (Table 1).

**Table 1:** Bacterial 16S amplicon sequencing of karst bulk soil and *C*. *henryi* roots and leaves.

Sample name	Number of Tags	Number of OTUs
1L	50924	446
2L	51322	352
B	37506	2519
1R	44226	780
2R	37898	835

Note: 1 and 2 are two sample plots. R and L represent the roots and leaves of C. henryi, respectively. B indicates the karst bulk soil. Tag is the assembly of overlapping reads. Tag sequences with a similarity no less than 97% are clustered into an OTU.

**Fig 1:**
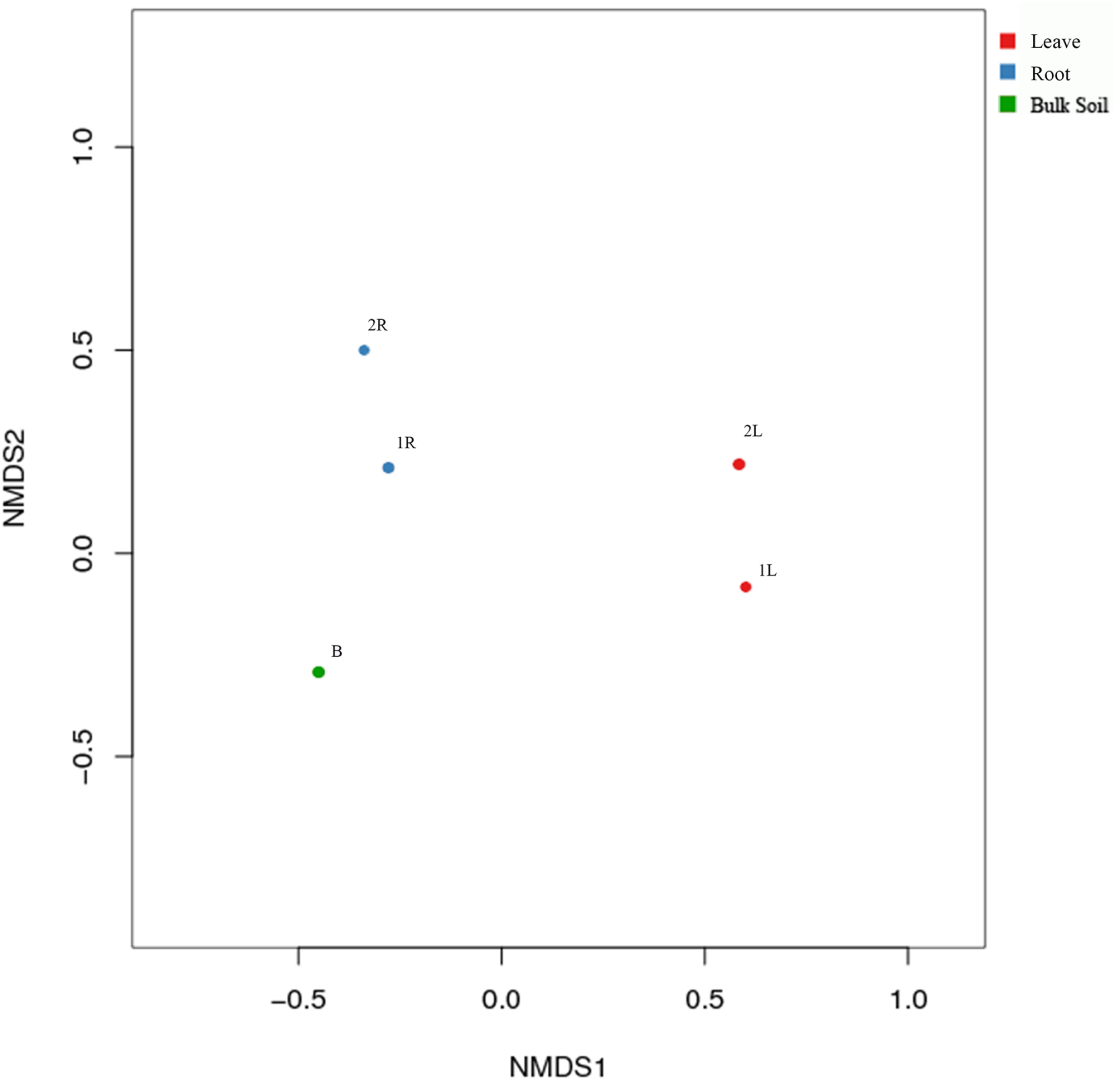
Sample compartments drive the bacterial composition. The figure shows non-metric multidimensional scaling (NMDS) analysis based on phylogenetic relationship, number and distance matrix. The horizontal and vertical axes represent the distance and each point indicates a sample.

By conducting the NMDS analysis, the relationship of the 5 samples was clearly displayed. In NMDS graph, closest samples had the minimum difference in bacterial community structure. The two leave samples of *C*. *henryi* were close to each other but far away from roots and soils. The two root samples of *C*. *henryi* were also close to each other, indicating that *C*. *henryi* was selective to endophytes. *C*. *henryi* selected certain bacteria to reside in its roots, and others to reside in its leaves. In other words, *C*. *henryi* and endophytes closely interact with each other (Fig 1).

### Selective enrichment of specific bacteria in the roots and leaves of *C*. *henryi*

**Fig 2:**
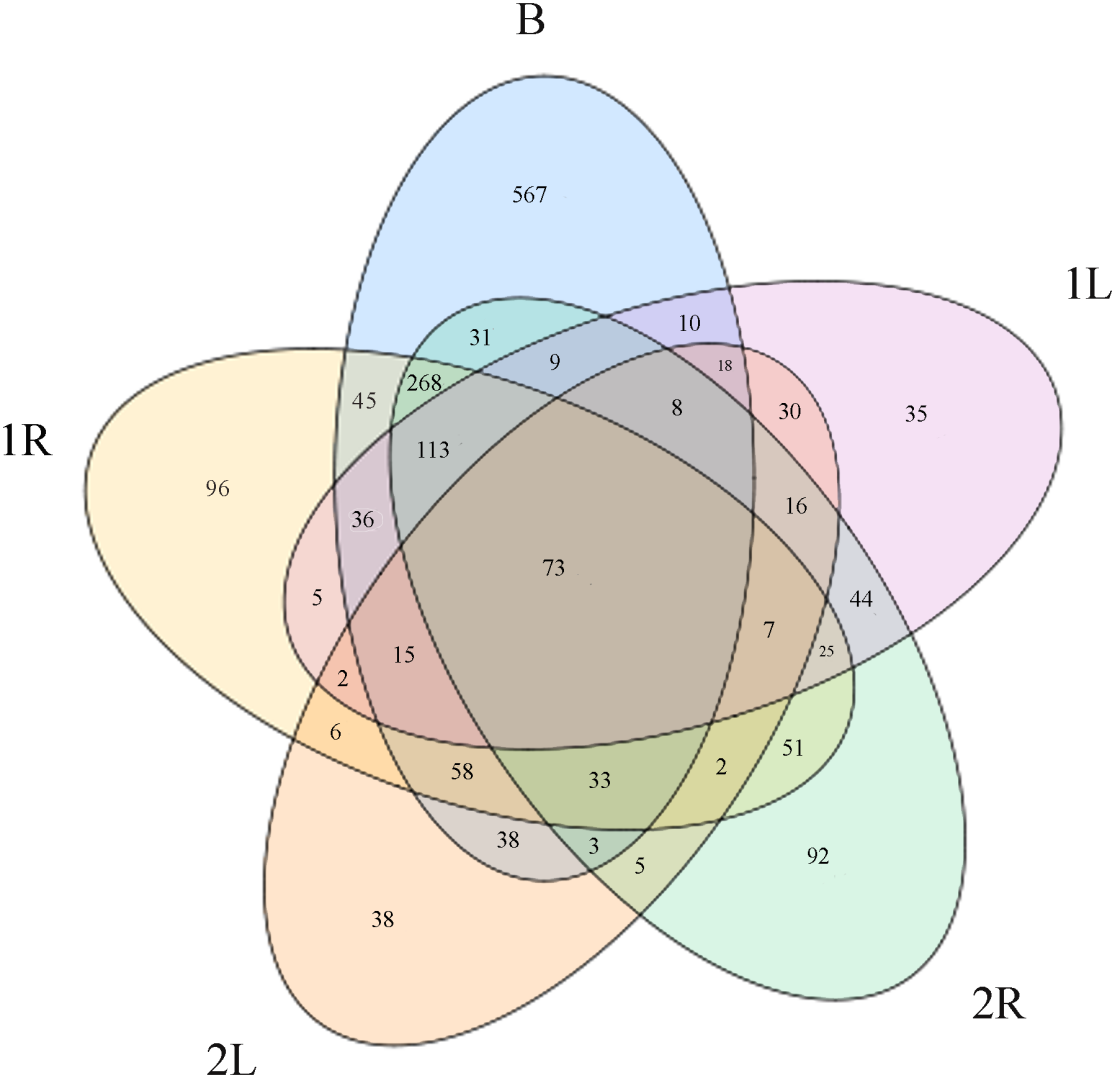
Venn diagram showing shared and specific OTUs among karst soils and roots and leaves of *C. henryi*.

By using R language to construct Venn diagram, the shared and specific OTUs of each sample could be clearly displayed (Fig 2). The bacterial communities in the same compartment (roots and leaves) of *C*. *henryi* between the two sampling plots in high calcium karst environment showed certain differences. The physical and chemical properties of soils were consistent between these two sample plots (S1 Table). Due to the close distance (5 meters), the climate and environment for *C*. *henryi* growth in these two sampling plots were also practically the same. Therefore, observed differences were probably caused by different genetic background and, to a lesser extent, by the developmental stages of *C*. *henryi* seedlings. The differences in bacterial community structures between both plots were lesser than those between different compartments (bulk soil, roots and leaves of *C*. *henryi* seedlings). Through Venn diagram analysis, 73 OTUs (GenBank accessions numbers MF114128 to MF114200) shared by karst bulk soils as well as roots and leaves of *C*. *henryi* from the two sample plots were identified (Fig 2). The bacteria represented by these 73 OTUs possibly play a key role in the adaptive capacity of *C*. *henryi* to karst high calcium soils.

**Fig 3:**
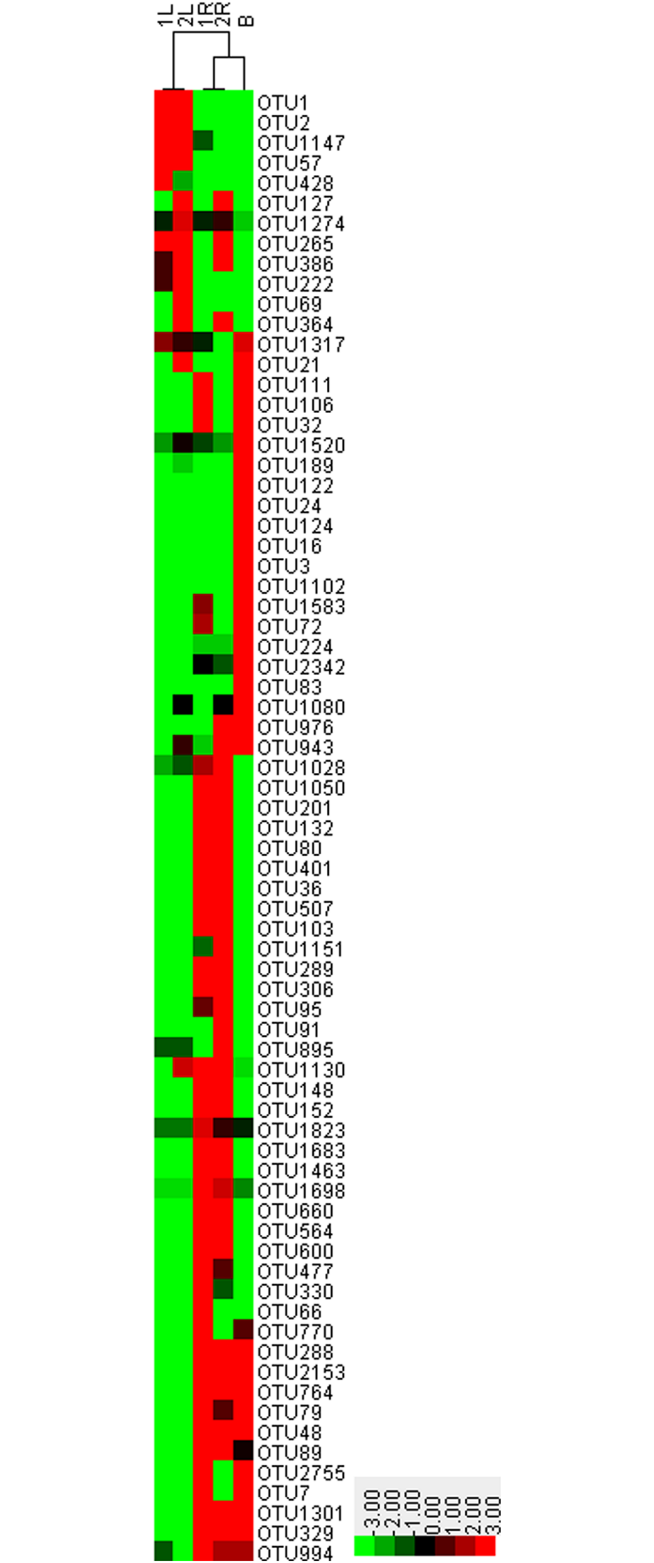
Abundance of core OTUs shared by karst soils as well as roots and leaves of *C. henryi*. In the Figs 1 and 2 are the two sample plots. R and L indicate the roots and leaves of *C. henryi*. B represents the karst bulk soil. Different sampling sites (sample plots 1 and 2) slightly influenced OTU abundance, while there was significant difference in the core OTUs abundance in different compartments (roots (R), leaves (L) and karst bulk soil (B)).

Although these 73 OTUs were present in all of the five samples, their abundances were significantly different (Fig 3). The differences between sampling sites (two sample plots) slightly influenced OTU abundances, while the abundance of core OTUs showed large differences among different compartments. The amount of OTUs such as OTU1, OTU2, OTU57 and OTU1147 in leaves of *C*. *henryi* was much higher than that in *C*. *henryi* roots and karst bulk soils. Similarly, *C*. *henryi* roots showed higher abundance of OTU1050, OTU201 and OTU132 than that in leaves and soils. The physical functions of OTUs showing different abundances in different compartments is an important problem worth exploring.

### Core OTUs showed different phylogenetic diversity from that of the overall OTUs

**Fig 4:**
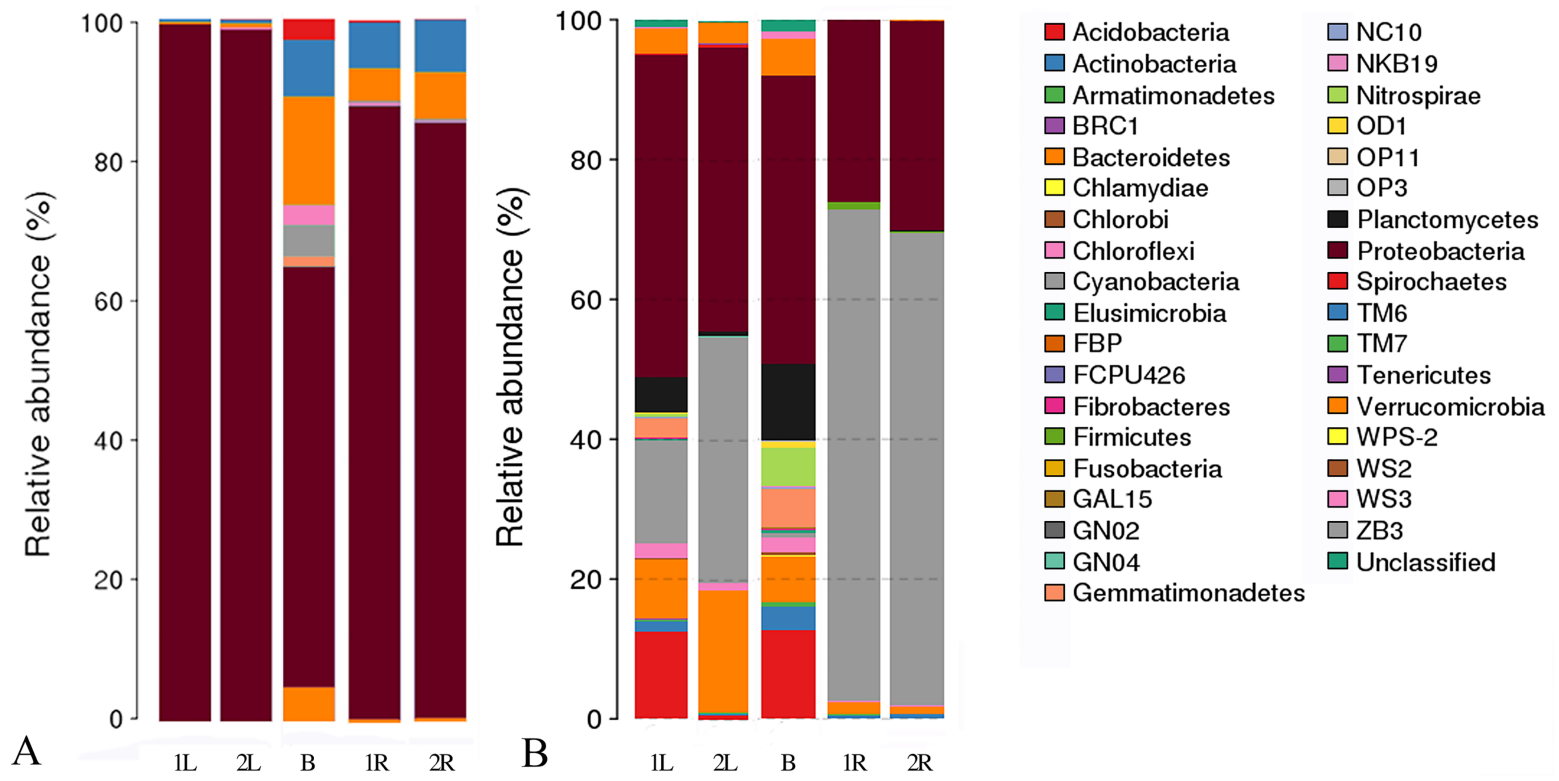
The phylogenetic diversity and structure of core OTUs (A) and overall OTUs (B) in karst soils as well as roots and leaves of *C. henryi*. 1 and 2 represents two sample plots. R and L indicate the roots and leaves of *C. henryi* respectively. B stands for the karst bulk soils.

The phylogenetic diversity of core OTUs (Fig 4A) differed greatly from that of the overall OTUs (Fig 4B). OP3 bacterium constituted an important part of *C*. *henryi* roots and leaves endophytes, but was rarely seen in core OTUs. In contrast, proteobacteria accounted for the vast majority of the core OTUs. The phylogenetic diversity and structure of core OTUs in *C*. *henryi* roots and leaves also showed significant differences. In *C*. *henryi* roots, proteobacteria were the most abundant, followed by actinobacteria and bacteroidetes. Moreover, in *C*. *henryi* leaves, the OTUs were completely dominated by the proteobacteria (Fig 4A).

### Multiple core OTUs showed high similarity with known stress-tolerant bacterial strains

**Fig 5:**
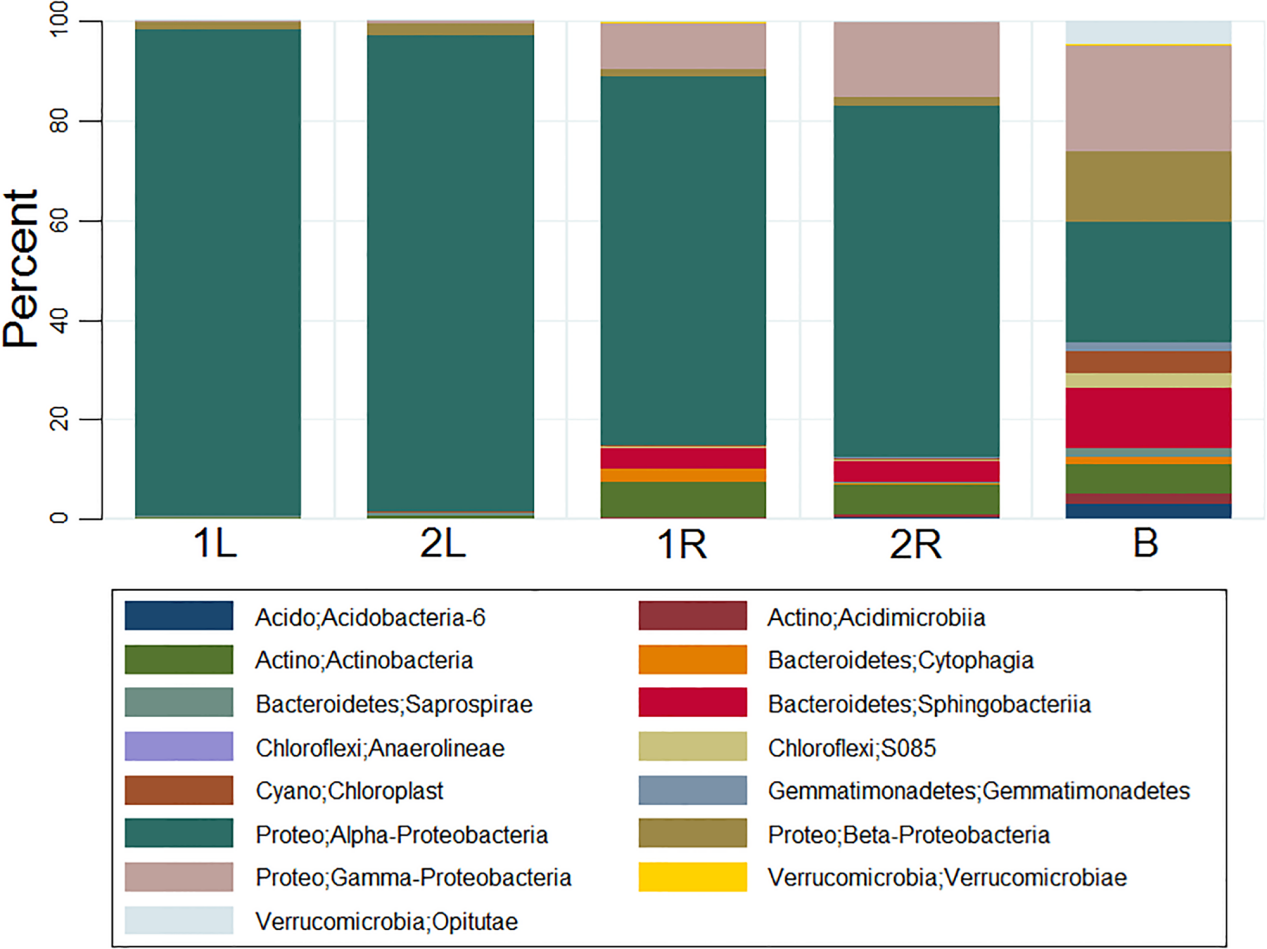
The taxonomy diversity and structure of core OTUs in karst soils as well as roots and leaves of *C. henryi* that are calcium stress-tolerant. 1 and 2 represents two sample plots. R and L indicate the roots and leaves of *C. henryi* respectively. B stands for the karst bulk soils.

Blast analysis (Nucleotide Blast in NCBI) of core OTUs showed that the 16S rDNA sequences of at least 5 core OTUs showed very high similarities with those of known calcium stress-tolerant bacteria. The results (Fig 5) indicated that they fell into acidobacteria, actinobacteria, cyanobacteria, proteobacteria, Bacteroidetes, chloroflexi, gemmatimonadetes and verrucomicrobia. The alpha proteobacteria were the most abundant in all samples (Fig 5). The abundance of alpha proteobacteria in leaves was higher than that in roots, which was also higher compared to soil samples. In addition, the gamma proteobacteria were abundant in the soil and root samples but rare in the leaf samples. The other stress-tolerant OTUs were more abundant in *C*. *henryi* roots and karst high calcium soils. They include Sphingobacteres (Bacteroidetes) and Verrucomicrobiales (Verrucomicrobiae).

### Functional profiles of core OTUs and overall OTUs were different

**Fig 6:**
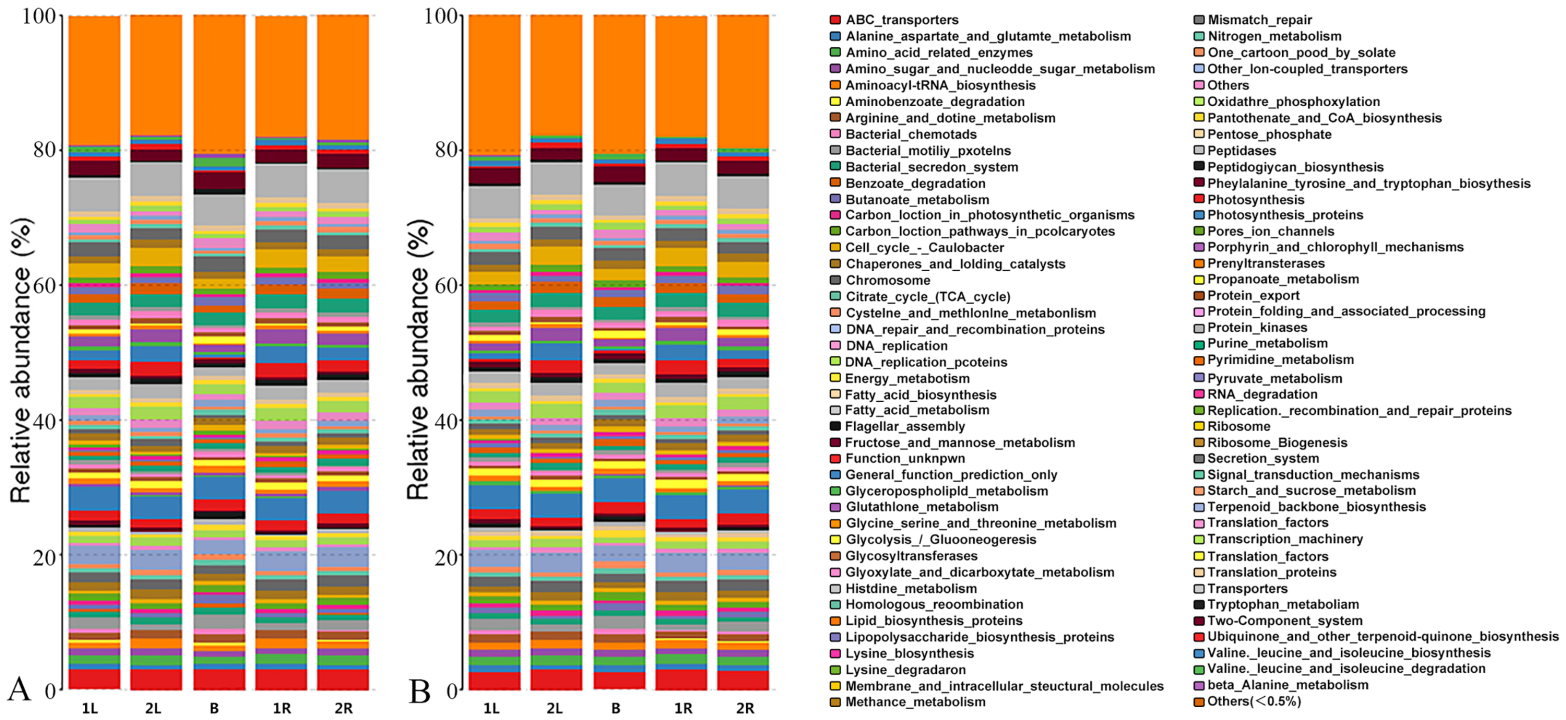
Gene functional profiling of core OTUs (A) and overall OTUs (B) in karst bulk soils as well as roots and leaves of *C. henryi*.

The PICRUSt software that can predict the functions of bacteria without utilizing metagenomic sequencing was used for functional annotation of core OTUs and overall OTUs. Most identified functional spectra were present in both overall and core bacteria. The predicted functions included ABC transporters, Mismatch repair, Glutathione metabolism, and Chaperones and folding catalysts relating stress responses, as well as Energy metabolism, Fatty acid metabolism, Glycolysis/Gluconeogenesis, Glycosyltransferases, Glyoxylate and dicarboxylate metabolism relating basic metabolisms. Fig 6 shows the relative abundances of level 1 KEGG Orthologs (KOs) functions of overall and core bacteria in the five samples. Gene functional compositions demonstrated significant differences in karst high calcium soils as well as roots and leaves of *C*. *henryi*. The difference in compartments was the decisive factor affecting gene functional profiling of bacterial communities, which indicated that the differences of gene functions of bacterial community were related to diverse living environments, namely high calcium soils, roots or leaves of *C*. *henryi*. In core bacterial community, the proportions of gene categories were comparable to those observed in overall bacterial community. This observation confirmed that the functional roles may be governed by core bacteria, though the interaction between these core bacteria and other OTUs is not to be neglected.

## Discussion

Researches have proved that there are various and abundant microbial communities in *Arabidopsis thaliana* [2, 3], crops (wheat, corn and rice) [21, 22] and economic crops (cotton and poplar) as well as in their rhizosphere [23, 24]. These microbial communities can greatly improve the adaptive capacity of host plants to adverse environments. The high content of calcium in karst soils is an important limiting factor for vegetation growth in karst ecological system. In this work, by sequencing the bacterial 16S rDNAs [25, 26], the compositions and characteristics of core bacterial communities relating to adaptive plants in high calcium environment were discussed.

In this research, the bacterial community structures in karst soils with high calcium content, as well as roots and leaves of *C*. *henryi* were quantitatively analyzed. Moreover, NMDS analysis was conducted to obtain the relationship between microbial communities in three different compartments. The bacterial community structure in leaves of *C*. *henryi* in the two sample plots showed a far distance from the roots and karst soils. In contrast, bacterial communities in high calcium soils and roots gathered together, indicating the existence of specific bacteria adaptive to high-calcium stress [27]. In leaves, the effects of host plants are the main determinant factors affecting bacterial community structures, while the selective biological pressures (host effects) and non-biology (high-calcium stress) jointly determine the bacterial community structures in *C*. *henryi* roots.

The current and previous researches indicate that there are hundreds of diverse microorganisms in the rhizosphere and the endophere of plant roots. Previous field studies demonstrated that the applications of individual bacteria strain cannot generally bring about the expected effects. This low efficiency of external inoculated strains is mainly caused by the competitions of the original microbial community in soils and plants. How to find bacterial strains that can stably infect and significantly influence host plants? To achieve this, several researchers have put forward a new strategy, that is, inoculating core microbial communities instead of individual strains [28, 29].

Determining the core microbial communities can help researchers to exclude temporary and instable plant-microorganism interactions and focus valuable research resources on bacterial strains that constantly and closely co-exist with plants and influence the phenotypes of hosts. By using Venn diagram analysis, 73 bacterial OTUs present in high calcium soils as well as roots and leaves of *C*. *henryi* were determined. In addition, the phylogenetic and functional characteristics of these 73 core OTUs were analyzed. The phylogenetic structures of core OTUs and overall OTUs were significantly different. According to the definition of core bacteria (bacterial strains shared by high-calcium soils as well as roots and leaves of *C*. *henryi)*, the bacterial strains represented by the core 73 OTUs were probably the key bacteria conferring to *C*. *henryi* its capacity to adapt to karst high calcium stress. These bacterial strains mainly belonged to the Proteobacteria phylum. In addition, by using high accuracy PICRUSt tool, the functions of overall and core bacterial communities were predicted. In core bacterial community, the proportions of gene categories related to osmotic and salt stress tolerance, including molecular chaperones, protein folding gene, β-alanine metabolism, and lysine biosynthesis, were larger than those in overall bacterial community. These findings revealed the molecular mechanisms underlying the adaptation mechanism of microbial communities to high calcium environment, and helped us find out some functional characteristics of microbial communities related to the adaptive capacity of the host plant. There were critical differences between the functional spectra of overall and core bacterial communities. The functional spectra of bacterial communities between different compartments showed greater differences. This further suggested that host plant, as a habitat of bacteria, significantly influences the bacterial community. It is worth noting that the PICRUSt results merely offer putative supporting information rather than direct evidences, because PICRUSt analysis cannot provide the actual quantity of gene expression [30]. However, analysis with the same procedures and standards can provide solid evidences for the differences of bacterial functional spectra among different compartments and between overall and core bacteria in high calcium karst habitat.

The key limitation of the present study was that the work was focused on the description of the bacterial strains in the karst soil and in the roots and leaves of *C*. *henry* which allowed the identification of the core OTUs and subsequent predictive functional profiling using PICRUST but did not experimentally scrutinize the actual relationship of core bacteria with *C*. *henry* and how those bacteria affect the host metabolic pathways. Thus, further well designed and focused studies on *C*. *henry* infection by the core bacteria and their related effects on the host are in the center of our future research goals.

In conclusion, this research provides novel insights for the structures and functions of soil microbial communities and endophytes of plants in high calcium adverse environment. Results indicated that *C*. *henryi* plants, commonly seen in high calcium adverse environment, adapt to high-calcium stress by closely interacting with endophytic microorganisms. Especially, potential beneficial functional bacteria were determined by prediction of core bacteria. Our findings are valuable in the sense that it helped us find microbial communities for field applications in karst ecological systems, and it will accelerate experimental studies aiming to determine microbial communities which can promote the adaptive capacity of plants to high calcium environment. There is no doubt that the integration of the top–down (surveying microbial communities based on pyrosequencing) and bottom-up (bacterial inoculation experiments) methods will better reveal the functional characteristics of microbial communities identified in this study. The present study showed substantial promises for sustainable agriculture in karst regions around the world.

## Supporting information

S1 Fig The rarefaction analysis was done with mothur program. (TIF)

S1 Table Karst bulk soils in Huajiang county are alkaline and infertile with high calcium content. The nutrient soils used as a control for comparison were for greenhouse use. Experiments were performed in triplicate and data presented as mean±sd (standard deviation).
(DOCX)
