# Supplementary figures and images for: Specialized core bacteria associate with plants adapted to adverse environment with high calcium contents
Source: PLoS One. 2018 Mar 8;13(3):e0194080. doi: 10.1371/journal.pone.0194080 (PMC5843345; doi:10.1371/journal.pone.0194080)

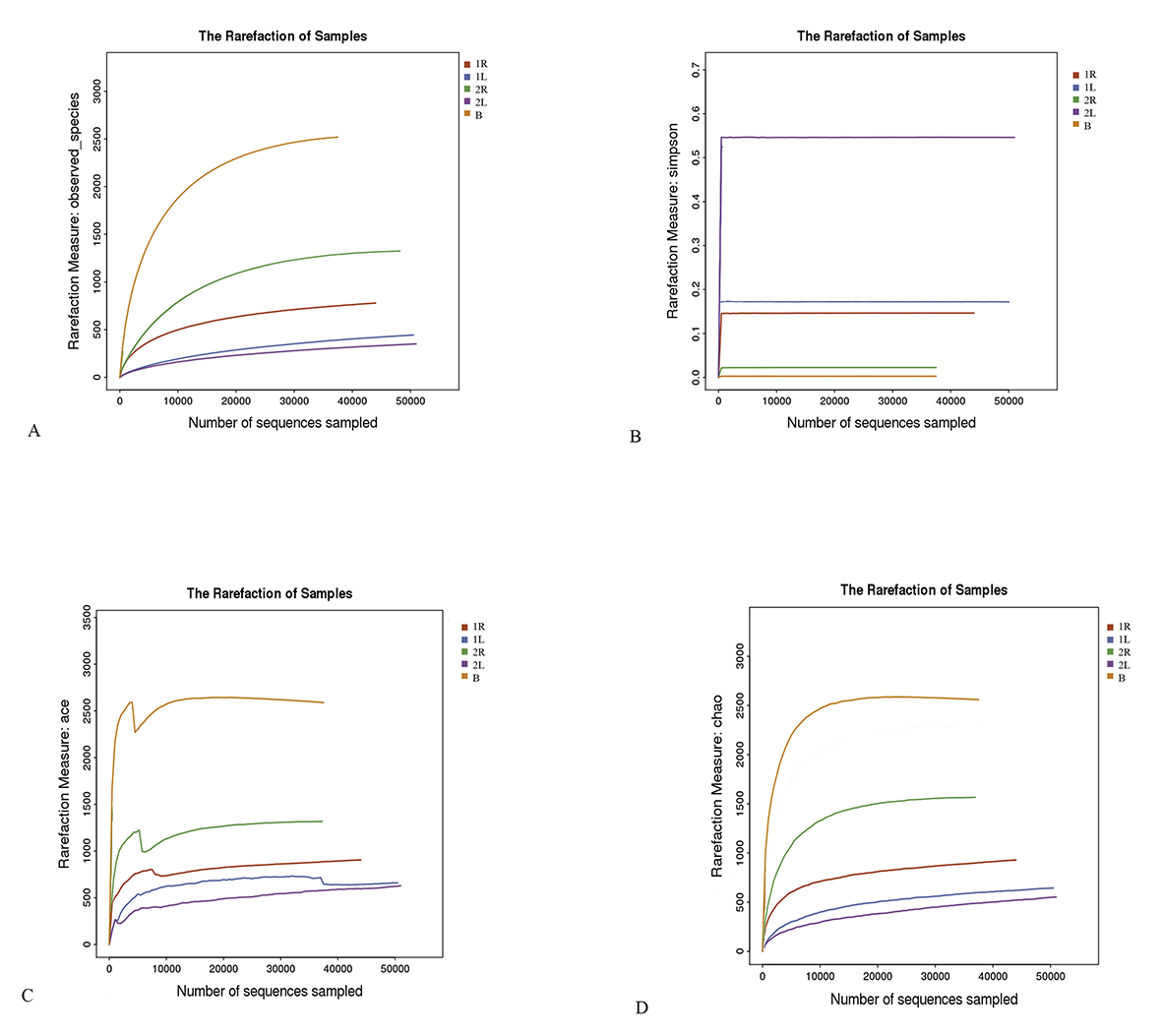

Supplement: S1 Fig — (TIF) [file pone.0194080.s001.tif]
