# Supplementary material for: Specialized core bacteria associate with plants adapted to adverse environment with high calcium contents
Source: PLoS One. 2018 Mar 8;13(3):e0194080. doi: 10.1371/journal.pone.0194080 (PMC5843345; doi:10.1371/journal.pone.0194080)
Supplement: S1 Table — The nutrient soils used as a control for comparison were for greenhouse use. Experiments were performed in triplicate and data presented as mean±sd (standard deviation). (DOCX) [file pone.0194080.s002.docx]

**S1 Table. Karst bulk soils in Huajiang county are alkaline and infertile with high calcium content.** The nutrient soils used as a control for comparison were for greenhouse use. Experiments were performed in triplicate and data presented as mean±sd (standard deviation.

| Soil Name | Total Minerals | | | | | | | | | | | | Carbon and Nitrogen | | | | | pH | |
| --- | --- | --- | --- | --- | --- | --- | --- | --- | --- | --- | --- | --- | --- | --- | --- | --- | --- | --- | --- |
|  | **P (%)** | **K (%)** | **Ca (%)** | **Mg (%)** | **S (%)** | **Zn (ppm)** | **Mn (ppm)** | **Fe (ppm)** | **Cu (ppm)** | **Al (ppm)** | **Na (ppm)** | **NH4 (ppm)** | | **NO3 (ppm)** | **Total C (%)** | **Total N (%)** |  | |  |
| Nutrient Soil | **0.05±0.009** | **0.10±0.015** | **0.24±0.018** | **0.13±0.04** | **0.03±0.006** | **58.66±2.13** | **493.55±224.84** | **9546.10±500.84** | **11.66±0.96** | **20439.00±** | **56.50±2.57** | **2.03±0.14** | | **34.10±3.10** | **2.66±0.49** | **0.21±0.017** | **6.20±0.35** | |  |
| Karst Bulk Soil  (sample plot 1) | **0.02±0.007** | **0.13±0.0023** | **2.28±0.12** | **0.32±0.05** | **0.02±0.003** | **77.30±5.2** | **560.07±30.5** | **6332.54±208.45** | **9.95±1.32** | **14139.87±375.85** | **49.42±1.98** | **1.34±0.63** | | **27.10±1.8** | **1.23±0.21** | **0.17±0.05** | **7.68±0.85** | |  |
| Karst Bulk Soil  (Sample plot 2) | **0.02±0.001** | **0.13±0.07** | **1.98±0.35** | **0.38±0.04** | **0.02±0.007** | **71.34±3.47** | **527.45±35.9** | **6068.65±175.39** | **7.77±0.86** | **15058.23±681.38** | **41.58±4.68** | **1.35±0.38** | | **23.82±1.58** | **1.28±0.16** | **0.17±0.09** | **8.02±0.24** | |  |
